# Standardized coronectomy versus total extraction for impacted mandibular third molars: a single-blinded prospective analysis of patient-reported outcomes

**DOI:** 10.3389/froh.2025.1647852

**Published:** 2025-08-05

**Authors:** Khalid Al-Ali, Roba Saqan, Sarah Alkhazraji, Kamis Gaballah

**Affiliations:** 1 Department of Oral and Craniofacial Sciences, College of Dental Medicine, University of Sharjah, Sharjah, United Arab Emirates; 2 Health Promotion Research Group, Research Institute for Medical and Health Sciences, University of Sharjah, Sharjah, United Arab Emirates

**Keywords:** third molar, impaction, surgical extraction, coronectomy, quality of life

## Abstract

**Introduction:**

Coronectomy is proposed as an alternative to surgical extraction for impacted mandibular third molars, particularly in cases with an elevated surgical risk of inferior alveolar nerve injury. However, this procedure is not widely adopted by many surgeons due to concerns about potential complications and the perception that patients may be less likely to accept this treatment option.

**Methods:**

This cross-sectional, prospective, single-blinded study compared patient-reported outcomes between standardized coronectomy and extraction of impacted mandibular third molars in 70 patients (aged 19–55 years) using the Postoperative Symptom Severity (PoSSe) scale.

**Results:**

While coronectomy avoided nerve injury, it resulted in relatively longer recovery times (40% vs. 28.6% requiring ≥5 days) and prolonged medication use (34.3% vs. 14.3% >5 days) compared to extraction. Coronectomy patients reported significantly higher pain and swelling scores, particularly among females (>25 years), though neither procedure adversely affected eating, speech, or quality of life. Gender and age influenced outcomes, with females and older patients experiencing more pronounced postoperative morbidity after coronectomy.

**Discussion:**

These findings underscore the need for demographic-specific counseling and tailored postoperative care when selecting coronectomy. Further research with larger sample sizes is recommended to validate these findings and optimize decision-making for mandibular third molar surgeries.

## Introduction

Mandibular third molars, often the last teeth to erupt in the dental arch, are the most commonly impacted teeth, with a reported prevalence ranging from 18% to 68.6% (1–4). Impacted third molars, whether partially or fully impacted, are associated with a range of pathological conditions, including pericoronitis, caries, cysts, and tumors. As a result, the extraction of both symptomatic and asymptomatic third molars is widely practiced. However, the proximity of mandibular third molar roots to the inferior alveolar canal (IAC) may pose a significant risk of inferior alveolar nerve (IAN) injury during extraction. The incidence of temporary altered sensations following extraction ranges from 1% to 5%, while persistent IAN involvement occurs in up to 0.9% of cases (5). Notably, over 30% of IAN injuries are reported in confirmed high-risk cases (6–8). In an attempt to minimize the risk of nerve injury, coronectomy has emerged as a viable alternative. This procedure involves removing the crown of the impacted tooth while leaving the roots intact, thereby reducing the likelihood of nerve injury (9, 10).

Understanding the advancements in extraction techniques, such as coronectomy, is crucial for clinicians to make informed decisions that optimize patient outcomes, recovery, and quality of life. Despite the availability of these surgical options, there is a notable gap in the literature addressing the patient's perspective on the recovery experience and perceived outcomes of coronectomy compared to total extraction. This lack of understanding limits clinicians' ability to guide patients effectively in choosing the most suitable treatment option. A notable gap exists in the literature, as no single-center study has directly compared standardized Patient-Reported Outcomes between coronectomy and extraction procedures. Previous reports have primarily focused on the outcomes of either procedure in isolation, with systematic reviews attempting to make indirect comparisons of postoperative complications based on various studies with inconsistent measuring and reporting criteria. This study aims to address this important clinical question by being the first to prospectively evaluate patient-reported outcomes following standardized coronectomy procedures performed by a single surgeon. Our study design minimizes technical variability and systematically captures patient-reported outcomes using well-validated measures throughout the immediate postoperative period. This study aims to compare the patient perspectives, recovery experiences, and patient-reported outcomes of coronectomy and total extraction of lower third molars. By analyzing these factors, the study seeks to provide a comprehensive understanding of the benefits and drawbacks of each procedure. The ultimate goal is to enhance clinical decision-making by integrating patient-centered insights into the management strategies for impacted third molars.

## Materials and methods

### Research ethics

The Dubai Scientific Research Ethics Committee approved the study with a reference code DSREC-SR-05/2023_01. Participation in the survey was entirely voluntary. All patients provided informed consent to participate in the study and were invited to a brief face-to-face interview during the stitch removal visit by one of the investigators. All research data were processed and stored in accordance with the institutional data protection regulations. Patient identification information was kept confidential.

### Study design

The study was cross-sectional, and the results were collected and analyzed from May 2024 to February 2025.

### Sample size calculation

To determine the required sample size, a power analysis was conducted. As there is currently no published study utilizing the PoSSe scale specifically for coronectomy procedures, the calculation relied on data from previous reports concerning patient-reported outcomes following the surgical extraction of impacted mandibular third molars 11–14. The primary outcome measure for this calculation was the PoSSe scale score. A two-sample independent t-test was chosen for the power analysis, assuming a two-sided test. Based on prior literature, specifically Zheng et al. (11), a clinically meaningful difference, i.e., effect size of 2.95 points on the pain subscore of the PoSSe scale, with a standard deviation of 3.33, was deemed essential to detect differences between the two groups. With a 5% significance level (alpha = 0.05) and 90% power (beta = 0.10), the calculation, performed using the PiFace software (http://homepage.stat.uiowa.edu/∼rlenth/Power/). The calculation determined that a minimum of 28 patients per group is required to detect the specified effect size. To account for potential lost follow-up such as missed appointments or withdrawals from the study, the sample size was increased by 25%. This adjustment brings the final target to 35 patients per group, for a total of 70 patients. This increase aligns with recommendations for clinical trials, which commonly experience a dropout rate of 10%–20%. Thus, it ensures sufficient statistical power even with minimal attrition. Using surgical extraction data for sample size estimation was necessary due to the lack of coronectomy-specific PoSSe studies. However, this approach remains valid because both procedures exhibit comparable postoperative symptom profiles.

### Research instrument

A previously validated Postoperative Symptom Severity (PoSSe) 11–14. The scale, a quality-of-life instrument, is designed explicitly for third molar surgery and has proven reliability, sensitivity, and responsiveness as a measure of the severity of symptoms after third molar extraction and the impact of these symptoms on the patient's perceived health. The PoSSe survey has seven subscales (Pain, Eating, Speech, Sensation, Appearance, Sickness, and interference with daily activities). The possible responses to each forced question are assigned a score. For each question, the answer scores could range from 0 to a number, which varies for each question. The scores of the responses to each question are summed to produce the overall PoSSe scale along with seven individual subscales.

### Participants and sampling technique

Patients included in the study are those who visited or were referred to the oral surgery clinic of KG for the total surgical removal and coronectomy of 1 or more mandibular third molars. Panoramic radiographs were taken to determine the need for surgical intervention, assess the difficulty of the extraction, and evaluate the potential risk of IAN injury. High-risk patients are those with one or more radiographic signs indicating the proximity of the mandibular third molar roots to the internal auditory canal (IAC) in their preoperative panoramic radiographs, as described by Rood and Shehab (12).

Patients with their third molar roots were closely related to the IAC. They were informed about the risks, benefits, and potential complications of both complete removal of third molars and coronectomy. After an adequate explanation of both procedures was provided, written informed consent was obtained. The patient underwent the agreed-upon surgical treatment option, and they were instructed to return for postoperative follow-up visits after 2 weeks. The same surgeon treated all patients with the same setup and facility. For this study, the patients were divided into two groups: the coronectomy and extraction groups.

### Surgical and research consent

The patient was informed about the impacted wisdom tooth, along with the proposed and alternative treatments. Additionally, the benefits of the proposed treatment, the risks associated with non-intervention, and the possible outcomes of the surgery are considered. In addition, the patients were informed that there is a risk of both early and late infections, which may necessitate further surgical procedures, including retrieval of the retained root piece if required. All patients signed a research consent form agreeing to participate in the study, which involved voluntarily answering questions about the surgery during the stitch removal visit two weeks after the surgery.

### Patient selection criteria

The selection of participants for this study was designed to ensure a homogeneous cohort of patients while minimizing confounding factors that could influence surgical outcomes. Inclusion criteria were set to identify patients with impacted mandibular third molars requiring either surgical extraction or coronectomy, who were otherwise healthy or with well-controlled systemic conditions (ASA I–II). Exclusion criteria aimed to eliminate high-risk individuals (e.g., ASA III+, heavy smokers) and those with comorbidities or treatments (e.g., immunosuppressants, radiation) that could impair healing and recovery or bias results. This approach enhances the internal validity of the study while reflecting real-world clinical decision-making.

### Inclusion criteria

Patients were included in the study if they met all of the following criteria: (1) Indication for surgical intervention: Patients requiring either Surgical extraction or Coronectomy of symptomatic impacted mandibular third molar, (2) Health status: Classified as ASA I or II (healthy or with mild systemic disease), (3) Age: 18 years or older, (4) Informed consent: Willingness to participate in the study and provide written consent, and 5) Surgical difficulty: Moderate difficulty based on the Pederson Difficulty Index.

### Exclusion criteria

Patients were excluded if they met any of the following criteria: (1) High anesthetic/surgical risk: ASA class III or higher (severe systemic disease or worse), (2) Heavy smoking: Consumption of >10 cigarettes/day (due to potential effects on wound healing), (3) Medications affecting healing: Current use of drugs that impair wound healing or immune response (e.g., immunosuppressants, chronic corticosteroids, bisphosphonates), (4) Radiation therapy: History of or ongoing radiation therapy in the head and neck region, and (5) Non-consenting patients: Unwillingness to participate in the study.

### Surgical procedures

All patients were given a preemptive analgesic (Oral soluble Ibuprofen 600 mg) immediately before the procedure. The local anesthetic technique, flap design, sectioning conditions of mandibular third molars, wound care, and postoperative medications were identical in both the coronectomy and total removal groups. Specifically, 1.8 ml of 2% lidocaine with 1:80,000 epinephrine was injected for the inferior alveolar nerve (IAN) block, along with 1.8 ml of 4% Articaine with 1:200,000 epinephrine for buccal mucoperiosteal infiltration. A developmental mucoperiosteal flap was elevated without a releasing incision, and minimal bone removal was performed for all patients.

In coronectomy Group patients, sectioning was initiated along the cementoenamel junction using a 1.6 mm fissure bur with a surgical straight handpiece at a speed of 40,000 rpm, an angle of approximately 25 degrees, and an average drilling depth of 9 mm, as described earlier. The cutting residual root surface was trimmed with a 4.2 mm round bur to equalize the sectioning level. Finally, after ensuring the cut margin was around 4 mm below the crest of both buccal and lingual alveolar bone margins, the wound was thoroughly irrigated with saline and sutured with two stitches of 3/0 polyglycolic acid. No additional pulp treatment or grafting was performed. Postoperative pain control involves prescribing Ibuprofen 600 mg soluble granules three times a day for 3 days with Paracetamol 1 g as rescue analgesics, and no antibiotics or medicated mouthwashes were prescribed. Postoperative instructions were explained to all patients. The duration of surgeries was 15–25 min. The stitches were removed from all patients 2 weeks after the procedure.

For Extraction Group patients, the impacted mandibular third molars were removed entirely using the same amount and technique of the local anesthetic. The teeth were accessed through the identical flap used in the coronectomy group. The teeth were sectioned using the same burs and handpiece, as well as the same speed, as the coronectomy group. The tooth sectioning aimed to remove resistance with minimal or no bone removal and unwanted pressure on the bone surrounding the nerve canal during elevation or up righting of the whole tooth. The surgery duration, wound care, closure, and postoperative medications, instructions, and follow-up were identical to those described in the coronectomy group. The recovery period was defined as the number of days until the patient reported no or little pain, swelling, or trismus.

### Blinding

The study maintained single blinding by using neutral group identifiers. The investigator (KA) responsible for interviewing the patients during the administration of the questionnaire and PoSSe scale was blinded to the surgery performed on the patients. The key linking group labels to their true identities was securely stored. It was only accessible to a designated member of the research team who was not involved in the analysis. Double blinding was not possible in the study settings, as the patient should agree to the type of the surgical procedure i.e., coronectomy vs. extraction based the potential risk of the nerve injury and provision of informed surgical consent.

### Data collection

Patients provided their responses independently, and the survey was answered, and co-author KA recorded all responses during the follow-up visit two weeks post-operatively.

### Data analysis

Data were analyzed using SPSS (Statistical Package for the Social Sciences) version 22 (IBM Corp., USA). The descriptive statistics for continuous data were reported as medians and interquartile ranges (IQRs), while for categorical data, frequencies and percentages were used. Differences in Periods of Recovery, Medication, and other specific postoperative symptoms (PoSSe) items between the two treatment techniques (extraction and coronectomy) by sex and age were assessed using the Chi-square test. The Mann–Whitney *U*-test was employed to compare overall PoSSe scores and its subscales between techniques by sex and age based on data normality. Simple and multiple linear regression analyses were conducted to identify factors associated with PoSSe levels. A *p*-value < 0.05 was considered statistically significant.

## Results

### Demographic data

The median age of the participants was 31 years, with an interquartile range (IQR) of 12.5 years and ages ranging from 19 to 55 years. Approximately three-quarters of participants (72.9%) were older than 25 years. Most of the participants were female, accounting for 72.9%. Regarding medical history, 85.7% of the participants reported no underlying medical conditions, and 14.3% had controlled Diabetes and were medicated for hyperlipidemia. The medication history was matched to the medical history, where more than 80.0% of participants were not on regular medications.

### Reasons for surgery

The reasons behind seeking surgical intervention, as analyzed using a chi-square test, are outlined in Table 1. Pain secondary to pericoronitis emerged as the most common cause overall, accounting for 79.9% of cases, with a higher prevalence among both groups. Caries on the third molar was the second most reported reason, observed in only 7.14% of cases. Other reasons, such as caries on the adjacent second molar and periodontal disease, were equal and accounted for only 3.4% of each reason. In terms of age, patients older than 25 years represented a larger proportion for all reasons. Pericoronitis-related pain was the primary treatment in 75.0% of patients aged over 25 years, while caries showed a similar trend, affecting 80% of patients in the same age group. In the extraction group, most were females with painful pericoronitis (70%) or males with carious teeth. Similarly, the coronectomy group consisted mainly of females with pericoronitis (73.3%), with 76.7% aged >25 years.

**Table 1:** The relationship between the reasons for the treatment and the type of surgery performed.

Group	Reasons for treatment		Pain (*n* = 30) (Secondary to pericoronitis)	Caries on tooth (*n* = 5)	Caries on adjacent tooth (*n* = 3)	Periodontal disease (*n* = 3)	Others (*n* = 2)
Extraction	Sex	Male	9 (30.0%)	2 (66.7%)	0 (0.0%)	0 (0.0%)	1 (50.0%)
		Female	21 (70.0%)	1 (33.3%)	1 (100.0%)	2 (100.0%)	1 (50.0%)
	Age Age	≤25 years	8 (26.7%)	1 (33.3%)	0 (0.0%)	0 (0.0%)	1 (50.0%)
		>25 years	22 (73.3%)	2 (66.7%)	1 (100.0%)	2 (100.0%)	1 (50.0%)
Coronectomy	Sex	Male	8 (26.7%)	1 (50%)	1 (50.0%)	0 (0.0%)	0 (0.0%)
		Female	22 (73.3%)	1 (50%)	1 (50.0%)	1 (100.0%)	4 (100.0%)
	Age	≤25 years	7 (23.3%)	0 (0.0%)	0 (0.0%)	0 (0.0%)	1 (25.0%)
		>25 years	23 (76.7%)	2 (10%)	2 (100.0%)	1 (100.0%)	3 (50.0%)

### Periods of recovery

A higher proportion of extraction patients (51.4%) recovered within 1–3 days compared to coronectomy patients (25.7%). Coronectomy patients reported relatively longer recovery times, with 34.3% requiring 3–5 days and 40.0% needing more than 5 days (*p* = 0.083). No significant age-based differences were observed (*p* = 0.313). However, the gender-stratified analysis revealed considerable variation among males: 50% of coronectomy patients recovered in 3–5 days, whereas none in the extraction group did. On the other hand, 45.5% of extraction patients reported taking more than 5 days to recover, compared to only 25.0% of coronectomy patients (*p* = 0.030) (Table 2).

**Table 2:** Postoperative recovery and medication intake periods by procedure type.

Category	Analysis	Time period	Extraction *N* (%)	Coronectomy *N* (%)	*p*-value
Recovery Period	Overall	1–3 days	18 (51.4%)	9 (25.7%)	0.083
		3–5 days	7 (20.0%)	12 (34.3%)	
		>5 days	10 (28.6%)	14 (40.0%)	
	Female	1–3 days	12 (50.0%)	7 (25.9%)	0.129
		3–5 days	7 (29.2%)	8 (29.6%)	
		>5 days	5 (20.8%)	12 (44.4%)	
	Male	1–3 days	6 (54.5%)	2 (25.0%)	0.030*
		3–5 days	0 (0.0%)	4 (50.0%)	
		>5 days	5 (45.5%)	2 (25.0%)	
Medication intake Period	Overall	1–3 days	19 (54.3%)	10 (28.6%)	0.054
		3–5 days	11 (31.4%)	13 (37.1%)	
		>5 days	5 (14.3%)	12 (34.3%)	
	Female	1–3 days	15 (62.5%)	8 (29.6%)	0.042*
		3–5 days	6 (25.0%)	9 (33.3%)	
		>5 days	3 (12.5%)	10 (37.0%)	
	Male	1–3 days	4 (36.4%)	2 (25.0%)	0.856
		3–5 days	5 (45.5%)	4 (50.0%)	
		>5 days	2 (18.2%)	2 (25.0%)	

* *p* < 0.05 indicates statistical significance.

Chi-square test used for all analyses.

### Medication duration

Medication use mirrored recovery trends, with extraction patients requiring shorter courses: 54.3% of patients used medications for 1–3 days, compared to 28.6% of coronectomy patients (*p* = 0.054). Coronectomy was associated with prolonged use (37.1% for 3–5 days; 34.3% for >5 days) compared to extraction (31.4% and 14.3%, respectively). Gender-stratified analysis revealed significant differences among females: 62.5% of extraction patients used medications for only 1–3 days, compared to 29.6% in the coronectomy group. In contrast, extended use (>5 days) was more frequent with coronectomy (37.0% vs. 12.5%; *p* = 0.042) (Table 2).

### PoSSe scale outcomes

The median of the overall PoSSe scale for all participants was 16.48. The median total PoSSe score was higher in the coronectomy group (19.33, IQR: 14.04) than in the extraction group (14.57, IQR: 15.89; *p* = 0.030). Among subscales, pain (coronectomy: 7.13 vs. extraction: 4.76; *p* = 0.018) and appearance, in the form of swelling (coronectomy: 3.00 vs. extraction: 0.00; *p* = 0.002) scores were significantly elevated in the coronectomy group. Other subscales (e.g., speech, sensation) showed no differences (all *p* > 0.05) (Table 3). Regression analysis identified procedure type as the sole significant predictor of total PoSSe scores (coronectomy *β* = 0.283, 95% CI: 0.674–13.715; *p* = 0.031). Age, gender, and comorbidities had no significant effects (all *p* > 0.05) (Table 3). Females in the coronectomy group reported higher total PoSSe scores (19.33 vs. 13.21; *p* = 0.039), pain scores (7.13 vs. 4.76; *p* = 0.008), and appearance scores (3.00 vs. 0.00; *p* = 0.022) compared to the extraction group. No such differences were observed in males (all *p* > 0.05) (Table 4). Age did not significantly influence PoSSe scores overall (*p* = 0.616). However, patients ≤25 years had higher appearance (swelling) subscale scores with coronectomy (3.00 vs. 0.00; *p* = 0.009) (Table 4). Pain duration of ≥5 days was more frequent after coronectomy (42.9% vs. 22.9%; *p* = 0.010), particularly in females (44.4% vs. 16.7%; *p* = 0.007) and patients over 25 years (38.5% vs. 24.0%; *p* = 0.047) (Table 5). The swelling was more common following the coronectomy (60.0% vs. 28.6%; *p* = 0.008), especially in females (63.0% vs. 33.3%; *p* = 0.035) and older patients (57.7% vs. 24.0%; *p* = 0.015) (Table 6).

**Table 3:** Posse scores and factors affecting outcomes.

Category	Variable		Extraction [median (IQR)]	Coronectomy [median (IQR)]	*p-value*
Total PoSSe Scores	Overall		14.57 (15.89)	19.33 (14.04)	0.030*
	Female		13.21 (16.36)	19.33 (21.39)	0.039*
	Male		16.45 (14.79)	17.40 (12.67)	0.600
PoSSe Subscales	PoSSe_Pain		4.76 (4.75)	7.13 (4.75)	0.018*
	PoSSe_Eating		5.25 (10.50)	5.25 (7.87)	0.362
	PoSSe_Speech		0.00 (0.00)	0.00 (0.00)	0.694
	PoSSe_Sensation		0.00 (0.00)	0.00 (0.00)	0.079
	PoSSe_Appearance		0.00 (1.50)	3.00 (4.50)	0.002*
	PoSSe_Sickness		0.00 (0.00)	0.00 (0.00)	0.131
	PoSSe_Interference		1.65 (1.37)	1.65 (3.03)	0.739
Factors Affecting PoSSe Scores	Age	≤25	10 (28.6%)	9 (25.7%)	0.616
		>25	25 (71.4%)	26 (74.3%)	
	Sex	Females	13.21 (16.36)	19.33 (21.39)	0.039*
		Males	16.45 (14.79)	17.40 (12.67)	
	Treatment Techniques				0.031*
	Medical Problems				0.089
	Medications				0.074
	Smoking				0.894
	Reasons for Treatment				0.383

* *p* < 0.05 indicates statistical significance.

Statistical tests used: Mann–Whitney *U*-test for comparisons, linear regression for factors affecting PoSSe scores. Median and interquartile range (IQR) are reported for PoSSe scores.

**Table 4:** Posse scores by gender and Age group.

Category	Category	Variable	Extraction median (IQR)	Coronectomy median (IQR)	*p-value*
Sex	Female	PoSSe Total	13.21 (16.36)	19.33 (21.39)	0.039*
		PoSSe Pain	4.76 (4.16)	7.13 (7.12)	0.008*
		PoSSe Appearance	0.00 (3.00)	3.00 (6.00)	0.022*
	Male	PoSSe Total	16.45 (14.79)	17.40 (12.67)	0.600
		PoSSe Pain	9.50 (9.49)	8.32 (2.38)	0.840
		PoSSe Appearance	0.00 (0.00)	2.25 (3.00)	0.051
Age	≤25 years	PoSSe Total	9.93 (11.88)	24.11 (9.19)	0.004*
		PoSSe Pain	4.76 (4.16)	9.51 (2.38)	0.013*
		PoSSe Appearance	0.00 (3.00)	3.00 (4.50)	0.072
	>25 years	PoSSe Total	16.26 (17.78)	16.07 (17.36)	0.486
		PoSSe Pain	7.13 (5.94)	7.13 (7.12)	0.219
		PoSSe Appearance	0.00 (0.75)	2.25 (3.38)	0.009*

* *p* < 0.05 indicates statistical significance. IQR, Interquartile Range.

Mann–Whitney *U*-test used for comparisons.

**Table 5:** Pain duration analysis by age and sex with the procedure type.

Category		Variable	Extraction *N* (%)	Coronectomy *N* (%)	*p*-value
Age	≤25 Years	No Pain	2 (20.0%)	0 (0.0%)	0.162
		1–4 Days	6 (60.0%)	4 (44.4%)	
		≥5 Days	2 (20.0%)	5 (55.6%)	
	>25 Years	No Pain	5 (20.0%)	0 (0.0%)	0.047*
		1–4 Days	14 (56.0%)	16 (61.5%)	
		≥5 Days	6 (24.0%)	10 (38.5%)	
Sex	Female	No Pain	6 (25.0%)	0 (0.0%)	0.007*
		1–4 Days	14 (58.3%)	15 (55.6%)	
		≥5 Days	4 (16.7%)	12 (44.4%)	
	Male	No Pain	1 (9.1%)	0 (0.0%)	0.677
		1–4 Days	6 (54.5%)	5 (62.5%)	
		≥5 Days	4 (36.4%)	3 (37.5%)	

*p < 0.05 indicates statistical significance.

Percentages represent within-group proportions. The chi-square test was used for analysis.

**Table 6:** Swelling analysis by age and sex with the procedure type.

Category	Variable	Category	Extraction *N* (%)	Coronectomy *N* (%)	Total *N* (%)	*p-value*
Age	≤25 years	No Swelling	6 (60.0%)	3 (33.3%)	9 (64.3%)	0.245
		Swelling	4 (40.0%)	6 (66.7%)	10 (35.7%)	
	>25 years	No Swelling	19 (76.0%)	11 (42.3%)	30 (58.8%)	0.015*
		Swelling	6 (24.0%)	15 (57.7%)	21 (41.2%)	
Sex	Female	No Swelling	16 (66.7%)	10 (37.0%)	26 (51.0%)	0.035*
		Swelling	8 (33.3%)	17 (63.0%)	25 (49.0%)	
	Male	No Swelling	9 (81.8%)	4 (50.0%)	13 (68.4%)	0.141
		Swelling	2 (18.2%)	4 (50.0%)	6 (31.6%)	
Overall		No Swelling	25 (71.4%)	14 (40.0%)	39 (55.7%)	0.008*
		Swelling	10 (28.6%)	21 (60.0%)	31 (44.3%)	

* *p* < 0.05 indicates statistical significance.

Percentages represent within-group proportions. The chi-square test was used for analysis.

## Discussion

We aimed to compare surgical coronectomy outcomes with total tooth extraction of the mandibular third molars in a controlled and standardized setting to eliminate the biased results that the authors often raise when reviewing various studies due to poor control of confounders. The study also provides a comprehensive comparison of the reasons for third molar surgery, highlighting significant differences influenced by regional practices, clinical priorities, and patient demographics.

In our study, pain secondary to pericoronitis was the most common reason for treatment. Similarly, several authors (13–16) identified pericoronitis as the leading indication for the extraction of impacted mandibular third molars, accounting for comparable proportions reported in our study. Conversely, Others (17) identified caries as the predominant reason for mandibular third molar extractions, representing 66.0% of cases, while pericoronitis accounted for only 18.5%. The higher caries rates may be attributed to dietary habits, oral hygiene practices, and limited access to early dental care. The participants in our study all have fully sponsored dental treatment, which may reflect their early presentation, mainly with pericoronitis, rather than delayed presentations, which are usually associated with more carious teeth and periodontal disease. Managing impacted mandibular third molars remains a critical challenge in oral surgery, particularly due to the risk of inferior alveolar nerve (IAN) injury. The patient reported outcomes following third molar surgical extraction were measured through various scales including the visual analogue scale and the oral health impact profile-14. Among those studies, recently, Starch- Jensen et al. (18) published a multicentric study of 12 European units including more than 400 patients and concluded that the surgical removal of third molar is associated with high treatment satisfaction and a relatively short period of discomfort. This study comprehensively compares coronectomy and total extraction, focusing on patient-reported outcomes using a validated postoperative symptom severity scale that was exclusively designed for the surgical extraction of mandibular third molars. We reported a much lower overall PoSSe score than the previous studies (11, 19–21). However, it is worth mentioning that all published reports investigating the PoSSe scale were based solely on patients who had undergone surgical removal of their impacted third molars, and none applied this scale to patients undergoing coronectomy. Although we reported relatively higher scores for coronectomy patients in the present study, the published scores were significantly lower than those reported for extraction patients in previous studies. In the present study, coronectomy effectively prevented IAN injury. However, patients who underwent this procedure reported a relatively prolonged recovery, higher pain scores, and increased swelling compared to those with their teeth extracted. These results align with those of Leung and Cheung (22), who noted delayed healing after coronectomy procedures but contrast with those of others (23), who reported lower pain in coronectomy patients. This discrepancy may stem from our standardized surgical protocol, which minimized variability. Importantly, these findings build upon our recent work (24), which established a universal sectioning depth (9 mm) and angle (25°) for coronectomy using imaging-based data. This protocol, adopted in the present study, ensures consistent surgical precision, reducing technical variability that often confounds outcomes in earlier research. This methodological rigor enhances the reliability of our morbidity data, particularly in terms of the observed differences in swelling and pain. Females reported worse outcomes after coronectomy, with higher pain and swelling scores, likely due to hormonally conditioned inflammatory responses (25). Older patients (>25 years) also experienced prolonged swelling, underscoring the need for age- and gender-tailored postoperative care. These findings align with those of other investigators (13) who noted higher PoSSe scores in females. Our study extends this by linking demographic factors to specific surgical techniques. To minimize the postoperative pain and swelling several reports have highlighted the role of intra and post operative steroid using various routes (26–28). Patients who underwent coronectomy required longer courses of medication, consistent with previous findings (29). In contrast, younger patients under the age of 25 tended to discontinue medications earlier, highlighting the influence of age on healing capacity (30). This study is the first to report the integration of our standardized coronectomy protocol with dedicated patient-reported outcomes for third molar surgery, addressing a critical gap in the literature. Prior studies lacked uniformity in surgical techniques, leading to inconsistent outcomes reported no pain differences (31), while others (22) found lower pain with coronectomy. By adopting a universal sectioning approach, we isolate the true morbidity associated with coronectomy, independent of technical variability. Additionally, our focus on high-risk cases, defined by radiographic proximity to the IAN, refines the comparative framework, unlike broader studies that included routine extractions. This specificity enhances the clinical relevance of our findings for surgeons managing complex impactions. The study's limitations include its single-surgeon design, which, while ensuring procedural consistency, may restrict the generalizability of the findings. While the calculated sample size provides adequate power for our primary outcome comparisons, it may be insufficient for robust subgroup analyses, which could potentially limit the statistical power of such exploratory analyses. However, it is crucial to note that our prospective and detailed patient-reported outcome study design differs fundamentally from retrospective investigations that report complication rates, as these typically benefit from larger sample sizes accumulated over extended periods of time. Some readers may see that the number of patients included was smaller than in some other retrospective or multicenter studies. It is worth noting that the nature of this report necessitated this number for the following reasons: the studies employed the PoSSe scale, which used comparable patient numbers but was limited to third molar extraction 11,13,14. This report was planned as a single-surgeon study to minimize the influence of various experiences and techniques on the surgery. The surgical procedures for both extraction and coronectomy, the duration of surgery, and post-operative management were standardized to minimize their impact on the outcome. Another confounding factor that influenced the sample size was the aim to include an equal number of patients undergoing extraction and those undergoing surgical coronectomy. The latter is typically offered to a small number of patients with problems related to mandibular third molars; therefore, we excluded more extraction cases than those for surgical coronectomy to ensure robust comparison and analysis. The follow-up was limited to two weeks as recommended by the design of the PoSSe scale and the standard weights on the subscores. This period focuses on reporting all acute problems encountered during the postoperative period. It is not intended to include late-onset complications of the coronectomy. Numerous retrospective and multicenter reports addressed the latter. Despite these limitations, coronectomy, when standardized, remains a safe alternative for high-risk third molars. However, it comes with trade-offs, such as longer recovery times and increased postoperative pain and swelling, necessitating careful patient selection. By integrating standardized coronectomy protocol, this study provides a reproducible framework for future research. Moving forward, we recommend emphasizing preoperative counseling to highlight demographic-specific risks, such as those faced by females and older adults, and implementing tailored postoperative care, including extended analgesia for coronectomy patients. Multicenter validation is also encouraged to assess the generalizability of our standardized approach and further refine clinical guidelines.

## Data Availability

The original contributions presented in the study are included in the article/Supplementary Material, further inquiries can be directed to the corresponding author.
