# Correction: Standardized coronectomy versus total extraction for impacted mandibular third molars: a single-blinded prospective analysis of patient reported outcomes

**DOI:** 10.3389/froh.2025.1682906

**Published:** 2025-11-06

**Authors:** Khalid Al-Ali, Roba Saqan, Sarah Alkhazraji, Kamis Gaballah

**Affiliations:** 1 Department of Oral and Craniofacial Sciences, College of Dental Medicine, University of Sharjah, Sharjah, United Arab Emirates; 2 Health Promotion Research Group, Research Institute for Medical and Health Sciences, University of Sharjah, Sharjah, United Arab Emirates

**Keywords:** third molar, impaction, surgical extraction, coronectomy, quality of life

**Author spelling error**

Author [Khalid Al-Ali] was erroneously spelled as [Mohamad Khalid Al-Ali].

Author contributions

KA-A: Conceptualization, Data curation, Validation, Writing – original draft, Writing – review & editing. RS: Data curation, Formal analysis, Investigation, Writing – original draft, Writing – review & editing. SA: Data curation, Investigation, Writing – original draft, Writing – review & editing. KG: Conceptualization, Data curation, Formal analysis, Investigation, Methodology, Project administration, Validation, Writing – original draft, Writing – review & editing.

Misspelled

The original version of this article has been updated.

